# Effect of over-the-counter whitening strips and toothpaste on shear bond strength of orthodontic brackets

**DOI:** 10.4317/jced.58099

**Published:** 2021-06-01

**Authors:** Brian Hohlen, Steven Makowka, Stephen Warunek, Thikriat Al-Jewair

**Affiliations:** 1 Orthodontist, Private Practice, Sioux Falls, South Dakota, USA; 2 Research Specialist, Department of Restorative Dentistry, School of Dental Medicine, State University of New York at Buffalo, Buffalo, NY, USA; 3 Clinical Assistant Professor, Department of Orthodontics, School of Dental Medicine, State University of New York at Buffalo, Buffalo, NY, USA; 4 Associate Professor and Graduate Program Director, Department of Orthodontics, School of Dental Medicine, State University of New York at Buffalo, Buffalo, NY, USA

## Abstract

**Background:**

To investigate: 1) the effects of over-the-counter white strip enamel bleaching and 2-Step whitening toothpaste application on the shear bond strength of orthodontic brackets at 24-hours and 7-days post-bleaching latent period; and 2) the correlation between amount of bleaching and shear bond strength.

**Material and Methods:**

Sixty human premolar teeth were randomly assigned into two bleaching groups, white strip group treated with Crest® 3D WHITE™ LUXE Professional Effects Whitestrips (Proctor & Gamble, Greensboro, NC), and whitening toothpaste group, treated with Crest® 3D WHITE™ BRILLIANCE 2-Step Toothpaste (Proctor & Gamble, Greensboro, NC). The groups were further divided into 4 subgroups A, B, C, D (n=15 per group). Subgroups A and C were bonded 7 days after bleaching while subgoups B and D were bonded 24 hours after bleaching. Differences in shear bond strength between the subgroups and an unbleached control group (n=15) were compared using the Kruskal-Wallis test. Spectrophotometric color changes were recorded pre- and post-bleaching.

**Results:**

The mean shear bond strength for the white strip groups were 10.35±3.6 MPa and 11.69±4.33 MPa for the 7-day and 24-hour delayed bonding periods, respectively. Means for the whitening toothpaste groups were 11.01±4.31 MPa and 10.83±3.79 MPa for the 7-day and 24-hour delayed bonding periods. The mean for the control group was 9.59±3.32 MPa. There were no significant differences in shear bond strength between the groups at 7-days and 24-hour (*P*=0.650). There was a significant difference between teeth bleached with white strips as compared to those bleached with toothpaste and controls. The correlations between color change and shear bond strength were not significant (*P*=0.448).

**Conclusions:**

There is a significant difference in the amount of whitening achieved by over-the-counter white strips compared to toothpaste; however, no significant effects on shear bond strength occurred after bleaching with white strips and toothpaste at 24-hours and 7-days.

** Key words:**Bleaching, shear bond strength, orthodontics, over-the-counter.

## Introduction

Numerous over-the-counter bleaching options are available to consumers including whitening strips, whitening toothpastes, and nighttime gels. At-home bleaching kits for night time use contain 5-15% carbamide peroxide as the active bleaching agent, whereas shorter duration daytime kits may contain 5-25% concentrations of hydrogen peroxide (1-7). As over-the-counter whitening products become available to consumers, questions arise regarding interactions of these products with various dental materials including orthodontic brackets bond strength.

In 1993, Bishara *et al*. (2) demonstrated that the use of 10% carbamide peroxide had no significant effect on the shear bond strength of orthodontic brackets. Bishara still recommended postponing the bleaching until after orthodontic treatment as it may aid in providing the maximal bleaching effect after all teeth are correctly aligned. In a later study, Bishara *et al*. (1) evaluated the effect of 10% carbamide peroxide (Opalescence bleach) and 25% hydrogen peroxide (in-office Zoom!) on the shear bond strength of orthodontic brackets. They concluded that a latency period of seven- or 14-days post bleaching with either an in-office or at-home bleach did not significantly alter the shear bond strength of orthodontic brackets to enamel.

In 1994 Miles *et al*. (8) demonstrated that patients must wait at least one week after using whitening products containing carbamide peroxide before having orthodontic brackets bonded. Their results indicated that there is a significant difference in bond strength between teeth bonded immediately after bleaching and those bonded one week after placement in distilled water. Conversely, Homewood *et al*. (9), reported that carbamide peroxide has no adverse effect on shear bond strength.

Uysal *et al*. (7) established that 35% hydrogen peroxide bleach has no net reduction in bond strength of orthodontic brackets. They concluded that the bleached groups had shear bond failure occur within the resin or at the resin and enamel interface; whereas the unbleached group had failures occur primarily at the bracket and resin interface. Patusco *et al*. (10) discovered that using 10% carbamide peroxide bleach has no significant effect on shear bond strength. However, 35% hydrogen peroxide bleach reduces the shear bond strength and decreases the total amount of composite resin remaining on the enamel surface after bracket debonding. The authors concluded that it is acceptable to orthodontically bond a patient 24 hours after use of 10% at-home carbamide peroxide bleach, but not after 35% in office hydrogen peroxide bleaching. Bulut *et al*. (11) found that bleaching with 10% carbamide peroxide immediately before bonding reduces the bond strength; however, they recommended treating the bleached enamel with sodium ascorbate or waiting a period of one week to reverse the decrease.

To our knowledge, there have been no studies to date about over-the-counter bleaching agents containing hydrogen peroxide and whitening toothpastes and their respective effects on shear bond strength of orthodontic brackets. It is also controversial if a latent period after bleaching should be recognized before orthodontic bonding, thus allowing enamel surfaces to remineralize and affording a more adequate resin bonding. The objectives of this study were to investigate: 1) the effects of hydrogen peroxide based over-the-counter white strip enamel bleaching and 2-Step whitening toothpaste application on the shear bond strength of orthodontic brackets at 24-hours and 7-days post-bleaching latent periods; and 2) the correlation between amount of bleaching and shear bond strength. We hypothesized that there is a difference in the bond strength between teeth bleached with whitening strips and toothpaste prior to bracket bonding and teeth that are not bleached prior to bonding; there is a difference in the bond strength between teeth that are bonded 24-hours versus one-week after bleaching; and there is an inverse correlation between shear bond strength and the amount of color change (bleaching).

## Material and Methods

This *ex-vivo* study was approved by the Institutional Review Board of State University of New York at Buffalo (#00001584). The inclusion criteria were human maxillary or mandibular first and second premolar teeth with sound buccal enamel surfaces. Teeth were excluded if they had visible caries, restorations, endodontically treated, subjected to storage chemicals or agents (i.e.: bleach), fractured or cracked, enamel malformation, decalcification, or hypocalcification visibly present.

-Sample size

A mean difference in shear bond strength of 5 MPs was considered clinically relevant. With a power of 90%, at least 13 teeth per group were needed.

-Study procedures

Seventy-five human premolars, collected from dental offices in Buffalo, New York, between June through October of 2017 were stored in 0.5% Chloramine-T, Trihydrate (Batch # 0000071525) for 1 week and then distilled water for 2 months prior to experimentation. All teeth were reduced to flat buccal enamel surfaces, embedded in acrylic cylinders and the exposed enamel was polished in sequence with 200, 400, and 600 grit sandpaper. Teeth were assigned random numbers then again randomly divided into five subgroups (A, B, C, D, and E) each containing 15 teeth. The groups were as follows:

• GROUP A: bleached with Crest Whitening strips and stored in artificial saliva for one week, then immediately bonded with an orthodontic bracket

• GROUP B: bleached with Crest Whitening strips and stored for 24 hours, then immediately bonded 

• GROUP C: brushed with Crest 3D Whitening toothpastes and stored in artificial saliva for one week, then immediately bonded 

• GROUP D: brushed with Crest 3D Whitening toothpastes and stored for 24 hours in artificial saliva, then immediately bonded 

• GROUP E: soaked in artificial saliva for 2 weeks (same time period as whitening application) as a control group 

All tooth whitening procedures were started at specified time intervals such that all groups completed their post-bleaching saliva bath on a coincident end date. Hereafter, all teeth were randomly selected for bonding with orthodontic brackets on the same day that shear debond tests were completed (day 21). All study procedures and measurements were conducted by one calibrated investigator (B.H.). On day 21, 10 representative samples (2 in each group) underwent scanning electron microscopy (SEM) testing to evaluate the enamel surfaces post-whitening.

-Bleaching procedures

White strip bleaching was conducted on 30 teeth using CrestCrest® 3D WHITE™ LUXE Professional Effects Whitestrips which contains 6.5% hydrogen peroxide (12). In Groups A and B, the whitening strips were applied to the exposed enamel surfaces for 1 hour per day for 14 consecutive days, as recommended by the manufacturer. All teeth were stored in artificial saliva at 37ºC between each new white strip application.

The second protocol utilized CrestCrest® 3D WHITE™ BRILLIANCE 2-Step Toothpaste on 30 teeth. Step-1 served as a “deep cleansing dentifrice” consisting of 0.454% stannous fluoride toothpaste and Step-2 is a “whitening finisher” containing a 3% hydrogen peroxide whitening gel (13). Teeth in groups C and D were brushed with Step-1 for 10 seconds and then wiped clean with a cloth before applying Step-2 and brushing for an additional 10 seconds. Exposed enamel surfaces were then cleaned and rinsed with copious amounts of distilled water. All teeth were then returned to the saliva bath and stored for 24 hours at 37ºC until the next application. The investigator was calibrated for brushing pressure and technique using an electronic gram scale to conduct the toothpaste application portion of the experiment. All saliva baths for bleached and unbleached teeth were changed every other day to minimize bacterial growth.

-Bonding procedures

Bonding procedures began using 37% phosphoric acid etch gel (3M Unitek, Monrovia, CA; LOT#: N893671) for 20 seconds followed by a thorough water rinse and 5 second air dry. A thin layer of Transbond MIP (3M Unitek, Monrovia, CA; LOT #: N862799) was applied with a microbrush, air dried, and light cured using a VALO Orthodontic Curing light (Ultradent Products, Inc. Jordan, UT). Seventy-five identical pre-pasted Victory Twin series mandibular incisor brackets (3M Unitek Monrovia, CA; LOT #: II2KF) were bonded with a uniform 300g force by way of a vertical mount, single rod jig. The average surface area of each bracket was 12.78 mm2. Brackets were cured for 5 seconds from the incisal, gingival, mesial and distal to attain 20 seconds total. A radiometer was used to test the VALO LED curing light immediately before bonding procedures (repeated three times) and a sufficient average reading of 780 mW/cm2 was recorded to ensure proper curing of all dental bonding materials. Immediately following bonding, all specimens were returned to a fresh saliva bath for a 30-minute soak to simulate an intraoral environment before bracket debonding tests were initiated.

-Study outcomes

1. Shear bond strength: An occlusogingival load was applied via an Instron universal testing machine (Model 33R4204, Norwood, MA, United States) with a flattened chisel end of a steel rod attached to the crosshead. All measurements were electronically recorded on a computer connected with the test machine that was set at a crosshead speed of 5 mm/min.

2. Spectrophotometric color differences: A spectrophotometric color measurement was completed using the VITA System 3D-Master VITA Easyshade® digital recorder prior to any bleaching procedures to record the baseline color. This same system was re-applied after bleaching for each group to determine the change in color (∆E). The CIE-L*a*b* system was utilized to record lightness (L*), green-red chromacity (a*) and blue-yellow chromacity (b*) (14). Color difference (ΔE) was calculated using the following formula: ΔE= [(ΔL*)2 + (Δa*)2 + (b*)2]1/2

3. SEM qualitative analysis: Scanning electron micrographs at X200 and X1000 magnifications were obtained on two specimens from each group (A-E) for a total of ten samples.

-Statistical analysis 

Intra-examiner reproducibility in colorimetry assessment was evaluated by Intraclass Correlation Coefficient (ICC) using six specimens by comparing differences in ΔE from pre-bleaching shade to post-bleaching. Descriptive statistics were calculated for each group. Tests of Normality using the Shapiro-Wilk test and homogeneity of variance using the Levene test were conducted. Analysis of variance (ANOVA) and Kruskal-Wallis nonparametric test where applicable were used to test for differences among the experimental groups for shear bond strength and for ΔE. Tukey’s Honestly Significant Difference (HSD) and pairwise Mann-Whitney tests were used for multiple group comparisons.

Pearson Correlation Coefficient was calculated to determine if there was a relationship between ΔE and shear bond strength. All tests were performed at a significance level of 0.05.

## Results

Intra-examiner reproducibility for colorimetry was 0.845 indicating a high degree of reproducibility (*P*=0.008).

-Shear bond strength

Results for the shear bond strength measurements are presented in Table 1. The means were similar for each group ranging from 9.59 Mpa for Group E to 11.69 Mpa for Group B. The Shapiro-Wilk tests indicated that the requirement of normality may be in question for Groups A (*P*< 0.029), D (*P*< 0.026) & E (*P*< 0.036). The Kruskal-Wallis test demonstrates there were no statistically significant differences (*P*=0.650) among the groups.

**Table 1:**
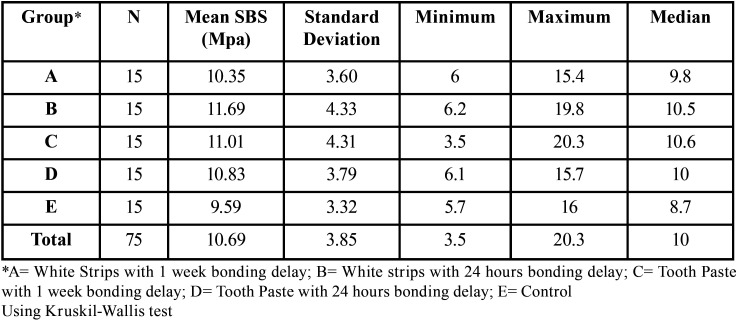
Mean shear bond strengths (SBS) for the bleaching and control groups.

-Color differences among the groups 

The mean ΔE ranged from 4.67 for group E to a 12.43 for group A, Table 2. The ANOVA test showed that there were significant differences among the mean color changes (*P*<0.001), Table 3. The Tukey HSD post-hoc procedure was that the experimental outcomes for change in color fall into one of two groups. The first group consisted of groups A and B, where a relatively large change in color was observed, while the second group (C, D, E) presented with relatively small changes in color. Groups A and B had similar medians. Groups C & E also appeared similar. Although group D looked different from the other groups it seemed more similar to C and E than to A and B based on the median.

**Table 2:**
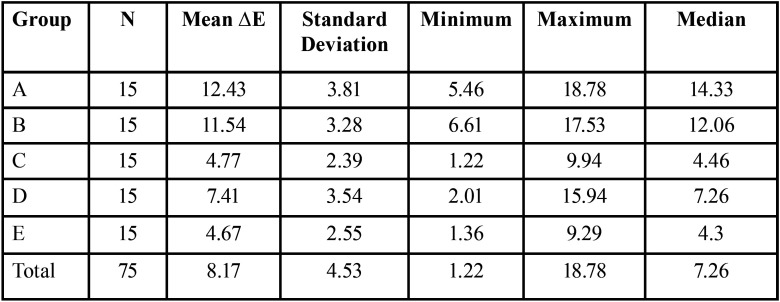
Mean color changes (∆E).

**Table 3:**
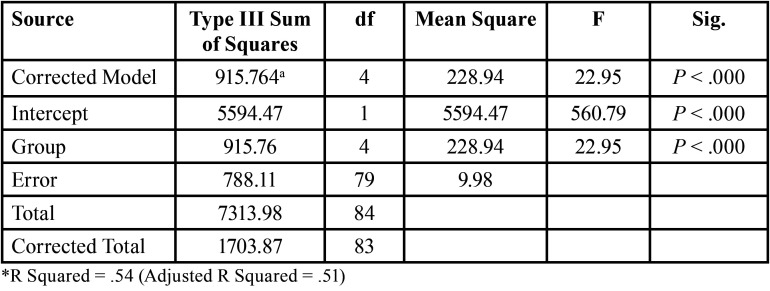
Results of ANOVA for ∆E.

**Figure 1:**
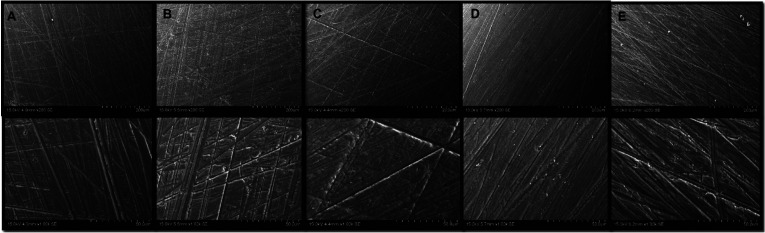
Scanning electron microscopy of groups A-E with 200 and 1000 magnification. Group A=White Strips (7-day); Group B= White Strips (24 hours); Group C=Toothpaste (7-day); Group D= Toothpaste (24 hours); Group E=Unbleached control. X200 magnification (upper row), X1000 magnification (second row).

No statistically significant correlations (*P*= 0.448) were found between color change and shear bond strength for groups A through E, Table 4. Comparing white strips Groups A and B to toothpaste Groups C and D, there was a trend demonstrating that as bleaching agent concentration decreases, bond strength increases; however, these changes were not statistically significant. Results from SEM are depicted on Figure 1. There were no observable morphological differences between the controls and the various bleached groups.

**Table 4:**
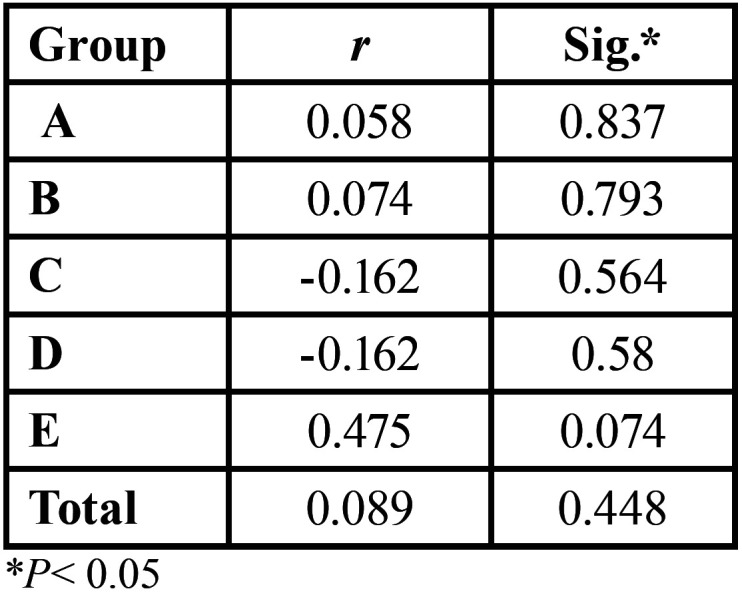
Pearson correlation coefficients of shear bond strength and ∆E.

## Discussion

With numerous over-the-counter bleaching products now available to consumers, dental patients are free to implement a bleaching regimen unsupervised by their dental professional. Inevitably, some patients employ these bleaching regimens immediately before beginning orthodontic treatment. With new over-the-counter bleaching products coming to market each year, clinicians are concerned with the effects of these agents on the enamel and dental bonding materials.

Many investigators have discovered that bleaching alters the superficial structure and microhardness of enamel, altering the prismatic form and leading to fewer resin tags and decreased bond strength (15-17). However, others observed no deleterious effects to the enamel surfaces (18). Some investigators have recommended resin bonding procedures be delayed for 2 or 3 weeks post-bleaching (1,19-21), yet numerous others did not observe significant variations in bond strengths of bleached versus unbleached enamel (1,2,7).

This study aimed to determine if there is a significant difference in shear bond strength between a 24-hour delay and a 1-week delay in bonding orthodontic brackets. To our knowledge, no previous studies have evaluated the following over-the-counter products: Crest® 3D WHITE™ LUXE Professional Effects Whitestrips and Crest® 3D WHITE™ BRILLIANCE 2-Step Toothpaste containing hydrogen peroxide concentrations of 6.5% and 3%, respectively. Most toothpastes are known to cause no changes in the enamel surface morphology as the mechanism of action of these toothpastes is more so dependent on the increased amount of detergents and abrasive particles rather than the low concentrations of peroxide present to lighten tooth color (22).

When the mean shear bond strengths of groups A through E was assessed, no significant differences were noted. Thus, the null hypothesis cannot be rejected. This is congruent with Akin *et al*. (23), Bishara *et al*. (1), and Patusco *et al*. (10) who each demonstrated no statistical difference between the bleached (10% carbamide peroxide) and unbleached groups. It is possible that bleaching with 6.5% hydrogen peroxide white strips and 3% gel toothpaste resulted in the same outcomes as bleaching with 10% carbamide peroxide. This may be due to the low concentrations of hydrogen peroxide present in both materials, as 10% carbamide peroxide breaks down into 3% hydrogen peroxide and 7% urea.

This study found a tendency for a decrease in shear bond strength as exposure to artificial saliva increases, mainly in the control group. This parallels the findings by Spyrides *et al*. (19) where storage in artificial saliva affected the control group, thus reducing its bond strength to 53% of the original. It is possible that the artificial saliva used as a storage medium contained components that form a “protective barrier” over the enamel surface, causing a decrease in microporosities and resin tags during bonding, thus decreasing bond strengths. This however warrants further investigation. Zeczkowski *et al*. (24) suggested the clinical situation can best be achieved using an “in situ “ storage medium which is essentially an intermediate stage between laboratory experimentation and clinical trials reproducing clinical conditions and conducting follow-up analysis outside of the mouth. Another factor that may compromise artificial saliva’s ability to create remineralization after bleaching is the presence of carboxymethylcellulose which is added to some formulations to increase its viscosity. Thus, the increased artificial saliva viscosity promoted by carboxymethylcellulose can decrease the rate of diffusion of minerals into the tooth structure (25,26).

In order for an orthodontic bracket to be clinically successful, Reynolds (27) determined it’s bond strength range should fall within or above the range of 5.9 – 7.5 MPa. All test groups in this study fell within this range, therefore, bonding after any of these bleaching procedures or time points should maintain acceptable clinical success levels. Mullins *et al*. (28) on the other hand determined that brackets bonded to recently bleached teeth (10% carbamide peroxide) have an increased chance of bond failure in both *in-vitro* and *in-vivo* studies. Mullins *et al*’s *in-vivo* study concluded that there should be a 2-week period between bleaching and bonding if no anti-oxidant agents are used.

When assessing the mean color change (∆E), there were statistically significant differences among the groups (*P*<.0001). According to Johnston *et al*. (14), clinically significant differences are represented by mean changes of ∆E >3.7. Interestingly, all groups receiving whitening products in this study as well as the control showed clinically significant changes in ∆E (>3.7). Color changes for groups A through D were expected. Further evaluation of Group E (control) however indicated a trend representing a gradual increase in the ∆E calculations up to 9.29 with no “jumps” to indicate an outlier. Interestingly, all remaining groups demonstrated this same trend.

Our findings are comparable to the results of Terezhalmy *et al*. (22) who demonstrated that whitening dentifrices remove surface stains by way of detergents and abrasive particles, and little to no bleaching of the enamel results from the low concentrations of peroxides present. The study reported that stain reduction with a whitening dentifrice is comparable to that seen after a dental prophylaxis. According to Zeczkowski *et al*. (24) the use of artificial saliva as a storage medium in enamel bleaching experiments reported the lowest values of change in brightness when compared to purified water, natural saliva, and in situ storage mediums.

The visualization of surface enamel on x200 and x1000 magnification SEM in this study showed no noticeable morphological differences between the controls and the various bleached groups. Ernst *et al*. (29) reported minimal to no morphologic changes occur to enamel while exposing enamel surfaces to bleaching agents containing 10% carbamide. On the contrary, Lai *et al*. (16) revealed an extensive difference in the etching patterns present on bleached versus unbleached enamel. Bishara *et al*. (1) demonstrated there is a visual difference in the uniform “honeycombed” appearance of bleached enamel as compared to unbleached enamel. It is possible our findings are related to the surface roughness that is present after sanding the samples with 200, 400 and 600 grit sandpaper. This assumption warrants further investigation.

Bonding orthodontic brackets to recently bleached teeth with over-the-counter hydrogen peroxide based bleaching strips and toothpaste does not appear to reduce bond strength. Therefore, bonding orthodontic brackets can be done after bleaching without the need to observe a latent period between bleaching and bonding. Several inherent limitations can be ascertained from this study. With an initial goal to standardize all samples and procedures, teeth were prepared to a flat enamel surface for bleaching and bonding procedures. In retrospect, this caused a roughened enamel surface that may have had an effect on bond strengths and also made comparisons of SEM unremarkable. As compared to previous studies, it is unknown which storage medium formulations for artificial saliva were used, thus making comparisons of result more challenging.

Future studies considering the effects of bleaching on orthodontic bracket bond strength may find it useful to consider the use of a standardized artificial saliva or use of an in-situ storage medium. Additionally, taking into account the Adhesive Remnant Index may aid in determining if bleaching increases the debonding of resin from enamel or if debonding is occurring between the resin and bracket pad interface, thus delineating more conclusive evidence of the effects of bleaching on bond strengths.

## Conclusions

1. There was no significant difference in orthodontic bracket bond strength between teeth that were bleached with white strips and toothpaste prior to bonding and those that were unbleached prior to bonding.

2. There was no significant difference in bond strength between teeth that were bonded 24-hours after bleaching with white strips or toothpaste and those that were bonded one week after.

3. There was no significant color change between teeth bleached with toothpaste and unbleached controls; however, there was a significant difference between teeth bleached with white strips as compared to those bleached with toothpaste and controls.
